# The 8p11 myeloproliferative syndrome: Genotypic and phenotypic classification and targeted therapy

**DOI:** 10.3389/fonc.2022.1015792

**Published:** 2022-11-03

**Authors:** Taotao Li, Gaoling Zhang, Xiaoling Zhang, Hai Lin, Qiuju Liu

**Affiliations:** ^1^ Department of Hematology, The First Hospital of Jilin University, Changchun, China; ^2^ Key Laboratory of Organ Regeneration and Transplantation of Ministry of Education, First Hospital, Jilin University, Changchun, China; ^3^ National-Local Joint Engineering Laboratory of Animal Models for Human Disease, First Hospital, Jilin University, Changchun, China

**Keywords:** EMS, FGFR1 rearrangement, targeted therapy, tyrosine kinase inhibitors, pemigatinib, infigratinib

## Abstract

EMS(8p11 myeloproliferative syndrome, EMS) is an aggressive hematological neoplasm with/without eosinophilia caused by a rearrangement of the FGFR1 gene at 8p11-12. It was found that all cases carry chromosome abnormalities at the molecular level, not only the previously reported chromosome translocation and insertion but also a chromosome inversion. These abnormalities produced 17 FGFR1 fusion genes, of which the most common partner genes are ZNF198 on 13q11-12 and BCR of 22q11.2. The clinical manifestations can develop into AML (acute myeloid leukemia), T-LBL (T-cell lymphoblastic lymphoma), CML (chronic myeloid leukemia), CMML (chronic monomyelocytic leukemia), or mixed phenotype acute leukemia (MPAL). Most patients are resistant to traditional chemotherapy, and a minority of patients achieve long-term clinical remission after stem cell transplantation. Recently, the therapeutic effect of targeted tyrosine kinase inhibitors (such as pemigatinib and infigratinib) in 8p11 has been confirmed *in vitro* and clinical trials. The TKIs may become an 8p11 treatment option as an alternative to hematopoietic stem cell transplantation, which is worthy of further study.

## 1 Introduction

8p11 myeloproliferative syndrome (EMS) or stem cell leukemia/lymphoma (SCLL), which is a very rare but aggressive neoplasm with the fibroblast growth factor receptor 1 (FGFR1) rearrangement on chromosome 8p11-12, is recognized as a distinct entity in 2016 World Health Organization (WHO) classification (1). In 2022 WHO classification, EMS belongs to myeloid or lymphoid neoplasms with eosinophilia and tyrosine kinase gene fusions (MLN-TK) (2). To date, fewer than 110 EMS patients have been reported worldwide. Most of them were male, occurring at any age. The disease progresses rapidly, usually into acute leukemia within one year. The molecular characteristics of EMS often involve chromosome 8 abnormalities, such as chromosome translocation, insertion, and inversion, which lead to the fusion of FGFR1 with partner genes to form any of 17 different fusion genes, resulting in the constitutive activation of tyrosine kinase (3). Because of its complex and diverse manifestations, EMS is often ignored or misdiagnosed as other hematological neoplasms such as aCML (atypical chronic myeloid leukemia) or CML. In the WHO classification, CML and aCML belong to myeloproliferative neoplasms, the distinction is that CML is defined as BCR-ABL1 fusion-positive resulting from t (9, 22) (q34; q11), while aCML is rare and characterized molecularly with BCR-ABL1 fusion-negative, and it is emphasized that accurate histological diagnosis has been to be the key to predict the prognosis of the disease (1, 4). To make the differentiation, it is necessary to detect BCR-ABL1 fusion-positive or FGFR1 rearrangements in peripheral blood (PB) or bone marrow (BM) by a combination of karyotype analysis, fluorescence *in situ* hybridization (FISH) and next-generation sequencing of molecular genetic techniques. In addition, the prognosis of EMS is very unfavorable. At present, only allogeneic stem cell transplantation (allo-SCT) improves the survival of these patients (5), but less than 50% of patients with EMS can undergo allo-SCT (6). Due to its resistance to traditional treatment, the drugs targeting tyrosine kinase inhibitors show the most promising results and have some advantages. This review will summarize the phenotypic and genotypic classification and the application of targeted therapy or EMS in the last 25 years, providing the latest data about the characteristics of this rare entity.

## 2 Genotypic and phenotypic classification

FGFR1 belongs to the fibroblast growth factor receptor (FGFR) family and is a member of the receptor tyrosine kinase (RTK) superfamily (7). At least four FGFRs have been found in the FGFR family, namely, FGFR1, FGFR2, FGFR3, and FGFR4. Their common structural feature is that they all contain extracellular immunoglobulin-like domains and cytoplasmic tyrosine kinase domains (8). The FGFR1 gene has a total length of 65 kb and contains 19 exons, which are located on the short arm of chromosome 8. The product encoded by the FGFR1 gene is a transmembrane protein located in the cytoplasmic membrane, which is divided into intracellular and extracellular regions. The extracellular domain is a signal peptide composed of an immunoglobulin-like domain I, an acidic box, a heparin-binding domain, and cell adhesion factor homologous domain, an immunoglobulin-like domain II, and an immunoglobulin-like domain III. The intracellular region consists of a near membrane domain and a tyrosine kinase domain (9). The acidic box in FGFR1 plays an important role in stabilizing its protein structure and ligand-receptor interactions (10). The molecular pathogenesis of EMS is characterized by FGFR1 rearrangement, which forms a fusion gene originating from translocation, insertion, inversion, or deletion (11), to genome variation, affecting FGFR1 mRNA transcription, and promoting the oncogenicity and genetic variation of the FGFR1 protein (12). FGFR1 fusion genes can be divided into two types: type I and type II. Type I refers to the FGFR1 gene located at the 3’ end of the fusion gene, and the FGFR1 tyrosine kinase domain is fused to the N-terminal oligomerization domain of the partner protein. The N-terminal oligomerization domain of the partner protein generates a fusion type protein that cannot bind to the FGF ligand and causes a conformational change in the FGFR1 tyrosine kinase domain (13). This stimulates the function of FGFR1 oncogene and constitutively activates its tyrosine kinase function, changes its localization, and subsequently activates PI3K-AKT, RAS/MAPK, STAT, and PLCγ/PKC in the downstream cell pathways to transmit abnormal signals (7, 14). The fusion genes of FGFR1 and its partners in EMS arise from type I. Type II is the opposite to type I, and the main difference is the fusion proteins retain the extracellular domain of FGFR1, which binds to FGF ligands, and is common in solid tumors (13). Even though the domains in the fusion proteins retained by FGFR rearrangement are different, in all cases the protein retains a complete kinase domain, suggesting that the kinase domain plays a vital role in the function of the fusion protein.

**Figure 1:**
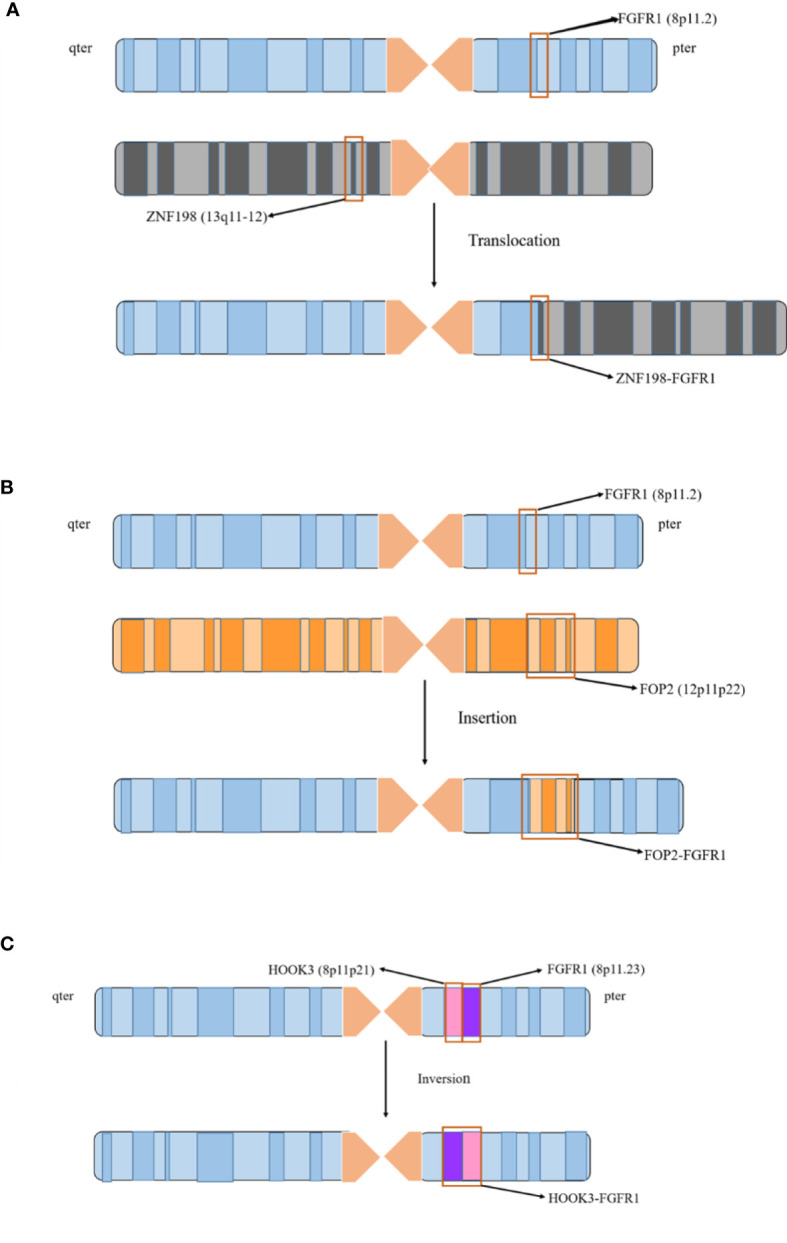
FGFR1 rearrangement involves three chromosomal abnormalities. 17 FGFR1 gene rearrangements existed in EMS, including 15 translocations, 1 insertion, and 1 inversion. **(A)** The 15 fusion genes are generated from chromosome translocation. It is illustrated by the case of ZNF198-FGFR1 to describe with which chromosome 8 and other chromosomes are formed by translocation. **(B)** FOP2-FGFR1 is the only rearrangement due to chromosome insertion. The FGFR1 gene on chromosome 8 and the FOP2 gene on the 12p11-p22 are breaking, and the dissociative FOP2 gene is reinserted into the FGFR1 gene fracture to form a fusion. **(C)** HOOK3-FGFR1 is a recently identified FGFR1 rearrangement in EMS, which is derived from chromosome inversion. The fragment between FGFR1 and HOOK3 genes located on the short arm of chromosome 8 is breaking, and the broken fragment is rotated 180 degrees, resulting in the fusion of HOOK3 and FGFR1.

Currently, it has been reported that 17 FGFR1 gene rearrangements exist in EMS, including 15 translocations, 1 insertion, and 1 inversion (**Figure 1**). Herein, we will further clarify the clinical characteristics of EMS with corresponding cases, and analyze the characteristics and functions of different FGFR1 rearrangements and partner genes (**Tables 1** and **2**).

**Table 1:** The characteristics and functions of different FGFR1 rearrangements and partner genes.

Genotypic	Chromosome abnormality	Year	Fusion site (partner;FGFR1)	Breakpoint (partner;FGFR1)	Oligomeric/dimeric domain of the fusion	Function of partner genes
ZNF198-FGFR1(15)	t (8;13)(p11.2;q11-12)	1998	exon 17;exon 9	exon 17;exon 9	Five zinc fingers or proline-rich domain (16)	DNA repair (17)
FOP1-FGFR1 (18)	t(6;8)(q27;p11.2)	1999	exon 5/6/7;exon 9	intron 6;intron 8	The LisH motif (18)	Anchoring of centrosome (19)
CEP110-FGFR1 (20)	t(8;9)(p11-12;q32-34)	2000	exon 15;exon 9	Intron x;exon 8	Leucine zipper (20)	Regulation of mitosis and cell cycle (21)
HERVK-FGFR1 (22)	t(8;19)(p11.2;q13)	2000	Not clear	Not clear	Not clear	Proliferation, transformation and tumorigenesis of normal cells (23, 24)
BCR-FGFR1 (25)	t(8;22)(p11.2;q11.2)	2001	exon 4;exon 9	intron 4;intron 8	Serine/threonine kinase domain (26)	Critical regulators of brain development (26)
NUP98-FGFR1 (27)	t(8;11)(p11.2;p15)	2001	Not clear	Not clear	The coiled-coil domain (not clear)	Nucleo-cytoplasmic transport (28)
FOP2-FGFR1 (29)	ins(8;12)(p11.2;p11p22)	2004	exon 4;exon 9	intron 4;intron 8	First two coiled-coil domains(30)	Not clear
TIF1-FGFR1 (31)	t(7;8)(q34;p11.2)	2005	Not clear	intron 11;intron 9	TRIM domain (31)	Transcription factor (32)
MYO18A-FGFR1 (33)	t(8;17)(p11.2;q25)	2005	exon 32;exon 9	Controversial	Presumably PDZ domain (34)	Unconventional myosin (34)
CPSF6-FGFR1 (35)	t(8;12)(p11.2;q15)	2008	exon 8;exon 9	intron 8;Not clear	RNA recognition motif (35)	3’ cleavage and polyadenylation of pre-mRNA (36, 37)
LRRFIP1-FGFR1 (38)	t(2;8)(q37;p11.2)	2009	exon 9;exon 9	Not clear	The coiled-coil domain (38)	Organism immune response
CUX1-FGFR1 (39)	t(7;8)(q22;p11.2)	2011	exon 11;exon 10	Not clear	Not clear	Tumor suppressor (40)
TPR-FGFR1 (41)	t(1;8)(q25;p11.2)	2012	exon 22/23;exon 13	intron 22;intron 12	TprMet, NPC relavant domain (41, 42)	Nuclear pore protein (43)
NUP358-FGFR1 (44)	t(2;8)(q12;p11.2)	2013	exon 20;exon 9	Not clear	Leucine zipper part (44)	Nucleo-cytoplasmic transport (45)
SQSTM1-FGFR1 (46)	t(5;8)(q35;p11.2)	2014	exon 9;exon 9	intron 8;intron 8	PB1 domain (46)	Regulating the activation of NF-κβ (46, 47)
TFG-FGFR1 (48)	t(3;8)(q12;p11.2)	2020	exon 8;exon 10	Not clear	Not clear	Not clear
HOOK3-FGFR1 (49)	inv(8;8)(p11.23;p11.21)	2022	Not clear	exon 11;exon 10	Not clear	Not clear

The basic information about the FGFR1 rearrangements are based on studies currently present in the literature.

**Table 2:** Number and common phenotypes of reported cases for EMS and the reported response for chemotherapy and TKIs.

Fusion	Number of cases	Common phenotypes	Physical and laboratory examination	Sensitivity to chemotherap	Numbers and results of allo-SCT	Sensitivity to TKIs
ZNF198-FGFR1	>30	T-LBL/T-lymphoma	Lymphadenopathy, hepatosplenomegaly, eosinophilia or monocytosis or both	Insensitive^1^	7,Remission;2,Recurrence	Sensitive (imatinib, MIDOSTAURIN)
FOP1-FGFR1	5	MPD, AML, B-ALL	Polycythemia without eosinophilia	Sensitive	No	Not tested
CEP110-FGFR1	>20	AML, T-LBL	Lymphadenopathy, purpura, skin lesions, eosinophilia and monocytosis	Insensitive	7,Remission;1,Recurrence	Sensitive (imatinib, dasatinib, pemigatinib)
HERVK-FGFR1	2	AML, SM-AHNMD	Polycythemia, poikilocyte, granulocytosis, abnormal megakaryocytes	Insensitive	1;Remission	Not tested
BCR-FGFR1	>30	CML, aCML, AML, B-ALL	Splenomegaly, eosinophilia	Insensitive	4,Remission;3,Recurrence	Insensitive (imatinib, dasatinib), Sensitive (ponatinib, pemigatinib)
NUP98-FGFR1	2	therapeutic AMML	Granulocyte hyperplasia with mononucleosis	Not tests	No	Not tested
FOP2-FGFR1	2	T-LBL, AML	Lymphadenopathy, eosinophilia	Sensitive^2^	No	Not tested
TIF1-FGFR1	5	CEL, AMML	Eosinophilia	Resistant^3^	No	Not tested
MYO18A-FGFR1	2	CML	Thrombocytopenia, monocyte, eosinophilic and basophil increased	Resistant	No	Not tested
CPSF6-FGFR1	1	Not reported	Lymphadenopathy and splenomegaly, neutrophils without eosinophilia	Resistant	No	Not tested
LRRFIP1-FGFR1	1	MDS, AML	Pancytopenia, eosinophilia	Not tests	No	Not tested
CUX1-FGFR1	1	pre-T-LBL	Neutrophils, lymphocytes and monocytes increased without eosinophils	Resistant	No	Not tested
TPR-FGFR1	4	AMML, AML-M5	Lymphadenopathy, increasing monocytes	Insensitive	1;Remission	Not tested
NUP358-FGFR1	2	MDS	Splenomegaly, a little eosinophilia	Sensitive	No	Not tested
SQSTM1-FGFR1	1	AMML	Neutrophils and monocytes increased, megakaryocytes	Not tests	No	Not tested
TFG-FGFR1	1	AML	Skin ecchymosis and splenomegaly, eosinophilia	Insensitive	No	Resistant (ponatinib)
HOOK3-FGFR1	1	MDS	Leukocytosis and thrombocytopenia.	Insensitive	No	Resistant (ponatinib)

The responsiveness to the TKIs and chemotherapy are based on the very few studies that have been reported so far; thus, the data included are not definitive. Addtionally, in many cases, the TKIs were used in conjunction with other chemotherapy or allo-SCT agents.

### 2.1 BCR-FGFR1/t (8, 22) (p11.2; q11.2)

FGFR1 is the second-most common partner gene of BCR (breakpoint cluster region) after the ABL (abelson leukemia virus) gene (26). Sequence analysis revealed that BCR exon 4 was fused in frame with FGFR1 exon 9, and genomic breakpoints occurred in intron 4 of BCR and intron 8 of FGFR1 (25). The BCR gene locus spans 130 kb and contains 23 total exons, with alternative exon 1 and exon 2; it will eventually encode a protein of approximately 1271 amino acids (50). Exon 1 encodes one serine/threonine kinase oligomerization domain, a growth factor receptor-binding protein 2 binding site (Grb2), the BCR-associated protein 1 interaction site (BAP-1), and two SH2 domains. Exons 3-8 encode a central ornithine exchange factor domain (GEF), and exons 19-23 contain the Racgap domain, as well as other PSD95, D1g1, and ZO-1 (PDZ) domain binding motifs (26, 51).

There is evidence that several domains of BCR play a crucial role in the pathogenesis of EMS. The fusion protein contains part of the RhoGEF (Rho ornithine exchange factor) domain of BCR, and it has kinase activity. The BCR-FGFR1 fusion causes a kinase-kinase fusion, it is where the tyrosine kinase domain of FGFR1 is fused to the serine-threonine kinase domain of BCR (13). A recent study pointed out that the GEF domain in BCR is related not only to the rapid onset of EMS with BCR-FGFR1 positivity but also to the phenotype of EMS disease progression. The deletion of the GEF domain leads to an increase in AKT activation by inhibiting the activation of RHOA and PTEN, accelerating the occurrence of leukemia, strengthening the survival and proliferation of cells, and promoting the proliferation of stem cells and lymph node metastasis (52). At the same time, BCR-FGFR1 also retains the coiled-coil domain of BCR (26).

To be worthy of our attention, Hu’s *in vivo* transplantation study demonstrated that microRNAs-17/92 are downstream effectors of FGFR1 in BCR-FGFR1-driven B-cell lymphoblastic leukemia (53). Moreover, the BCR-FGFR1 fusion protein also depends on the heat shock protein 90 (Hsp90) complex to escape the dissolution of the proteasome because BCR-FGFR1 acts as a client of chaperone Hsp90 (54).

Patients with BCR-FGFR1 fusion can have a similar presentation to BCR-ABL1 positive CML (55). In some case reports, it also tends to present AML-like and ALL phenotypes (56). Patients progressed rapidly into AML/ALL within one or two years of the diagnosis of EMS, but the relevant pathological mechanism is still unclear. Khodadoust and Morishige also reported three-line mixed phenotype acute leukemia with BCR-FGFR1 (57, 58). It is observed for phenotypic change in the course of the same patient. In previous research, it was proposed that the therapeutic drug blinatumomab may enhance the transformation of acute lymphoblastic leukemia into myeloid leukemia (59). Moreover, a meta-analysis of data from 20 patients indicated that BCR-FGFR1-positive cells might be derived from myeloid/B progenitor cells, but the mechanism determining the differentiation of B-myeloid cells is unclear (6).

### 2.2 ZNF198/ZMYM2-FGFR1/t (8, 13) (p11.2; q11-12)

ZNF198 was also described in previous reports as ZMYM2, FIM, and RAMP. ZNF198 is located at 13q11-12, and its orientation is from the telomere to the centromere. The ZNF198 gene is organized into 26 exons with an initiation codon located in exon 4, which is predicted to encode a 1377 amino acid nucleoprotein with five zinc fingers as the MYM domain (60), and it might participate in DNA repair by other proteins to form a complex (17).

ZNF198-FGFR1 fusion is the most frequent fusion type in EMS other than BCR-FGFR1. The 17 exons of ZNF198 are fused with 9 exons of FGFR1, as well as a breakpoint located at the same position in most cases. However, new studies have shown that the fusion is not only produced by a balanced translocation but also involves the insertion and internal inversion of the 13q11-12 chromosome (60–62); therefore, the breakpoint of the fusion is still not clear. The fusion gene produces a 152 bp transcript that is located in the cytoplasm, according to *in vitro* studies (15, 63). It is speculated that the fusion also encodes a protein with a length of 146 kDa containing approximately 1309 amino acids that is located in the cytoplasm (64). The fusion protein consists of five zinc fingers, a proline-rich domain, and FGFR1 the entire tyrosine kinase domain of FGFR1 (16). Exon 9 of FGFR1 encodes the tyrosine kinase domain; in this context, the zinc finger domain of ZNF198 is fused to the tyrosine kinase domain of FGFR1 (15, 64). There is an argument as to whether the carcinogenicity of the fusion comes from the oligomerization of the proline-rich domains or the dimerization of the zinc finger domains (16, 65). In short, the studies discussed above are basically *in vitro* studies, and more *in vivo* research is needed to confirm these ideas in the future.

There are at least 30 cases reported to be ZNF198-FGFR1 positive with EMS. At the time of onset, the diversity of clinical phenotypes and laboratory tests makes the diagnosis of the disease more difficult. Lymphadenopathy and hepatosplenomegaly are often the first symptoms. Most ZNF198-FGFR1-positive EMS patients are diagnosed with T-LBL/T-lymphoma (66). Cases involving B-ALL alone are relatively rare and are more common in cases of T/B double line involvement or acute mixed leukemia (67). The fusion suggests that the disease may originate from hematopoietic progenitor cells or stem cells and has the potential to differentiate along various lines. In a mouse model of ZNF198-FGFR1, T-cell receptors on the surface of tumor cells were found to have an α-deletion hindering the recruitment of CD3, preventing the maturation of CD4 (+)/CD8 (+) double-positive T cells, and upregulating BCL2, IL-7 receptor-α and IL-2 receptor-α in tumor precursor cells, allowing them to escape apoptosis in the thymus, which may be one of the reasons why this fusion more easily induces T-lymphocytic leukemia/lymphoma phenotype (68). The expression of ZNF198-FGFR1 is related to specific PAI-2 (plasminogen activator inhibitor-2/SERPINB2)-mediated anti-apoptosis, which is possibly one of the reasons for the high malignancy of leukemia cells (69).

### 2.3 CEP110-FGFR1/t (8, 9) (p11-12; q32-34)

CEP110, also known as CTNL, is located at 9q32-34. CEP110 comprises 19 exons spanning approximately 26 kb and is inferred to encode an acidic protein with 994 amino acids and a molecular weight of 110 kDa (20). The CEP110 protein binds to the centrosome *via* five repeated leucine zippers (L-X (6)- L-X (6)- L-X (6)-L), which are at amino acid positions 28-49, 98-118, 496-517 and 689-710, respectively (20). The position of CEP110 in the centrosome of mother and daughter is different (21), which might be related to the different roles of CEP110 in different stages of cell mitosis. CEP110 and nine other peptides could change the structure of the mitotic interphase centrosome, which is very important for the function of the microtubule-organizing center (21).

Because of a balanced translocation, CEP110-FGFR1 is a chimeric gene consisting of exon 15 of CEP110 fusing with exon 9 of FGFR1 (27), and its breakpoint is located in exon 8 of FGFR1 and an intron of CEP110. The N-terminal of CEP110-FGFR1 retains the leucine zipper of CEP110, the C-terminal contains the tyrosine kinase domain of FGFR1 in the cytoplasm, and the protein is approximately 150 kDa (20). The leucine zipper has dimerization potential in CEP110-FGFR1 and mediates the activation of constitutive tyrosine kinase activity. CEP110 is located in the centrosome but the fusion protein is in the cytoplasm, which may be relevant to the occurrence of EMS. CEP110-FGFR1 with EMS is the third most common phenotype, with nearly 20 cases. Although most patients present with lymphadenopathy, purpura and skin lesions are also common with such fusion genes (70). The bone marrow aspiration and peripheral hemogram of patients are often accompanied by eosinophilia and monocytosis. There is also a complex clinical phenotype, which is often characterized by myeloid leukemia. T-LBL is more common in enlarged lymph node biopsies.

### 2.4 FOP/FGFROP-FGFR1/t (6, 8) (q27; p11.2)

The FGFR1 oncogene partner, referred to as FGFROP and FOP, is located at locus 6q27. The whole FOP gene is 1630 bp and comprises 13 exons (71). This protein may encode a protein of approximately 44.3kDa, including 399 amino acids, with an α-helically folded conformation (71). The α-helical region contains (L-X_2_-L-X_3-5_-L-X_3-5_-L) leucine-rich repeats, including a Lish motif, which can dimerize (71). FOP is also in the centrosome and participates in forming an MT-anchored centrosome complex (19).

The in-frame fusion results from the 5/6/7 exons of FOP fusing to the 9th exon of FGFR1, and the breakpoint of the fusion is in intron 8 of FGFR1 and intron 6 of FOP, but it is changeable (18, 27). The FOP-FGFR1 fusion protein contains the N-terminal of the leucine-rich sequence of FOP (retaining the LisH motif) (18), and while it is in the centrosome, the fusion interferes with the normal function of FOP. In addition, the fusion could also protect cells from apoptosis by regulating BCL2 (B-cell lymphoma-2) overexpression and Caspase 9 inactivation (72, 73). Furthermore, the fusion proteins target the centrosome, activate the signaling pathway of this organelle by promoting centrosome phosphorylation, and continuously participate in the regulation of the cell cycle so that cells may overcome G1 blockade and obtain the ability to proliferate and survive (74). There is a possibility that the poor proliferation signal transduction of the fusion protein may depend on its abnormal localization and dimerization, and FOP is likely to be the cause of both (75).

Cases of FOP-FGFR1 fusion are rare, and only 5 cases have been reported. Among them, three cases are incomplete due to the early stage and inconsistent diagnostic criteria at that time. It is noteworthy that three of the cases were accompanied by polycythemia vera, without eosinophilia in the bone marrow and hemogram, which might also be due to the lack of records (18, 76). This finding supports the hypothesis that the FOP gene plays an important role in the proliferation and differentiation of erythroid cells (18). MPD (myeloproliferative diseases), AML, and B-ALL were included in their clinical phenotypes, but the complete characterization of the fusion was limited by the scarcity of cases.

### 2.5 NUP98-FGFR1/t (8, 11) (p11; p15)

NUP98 is situated on 11p15.4, 3.9 Mb from the telomere, and it consists of 33 exons, producing a transcript 122 kb in size that codes for 1729 amino acid residues of the NUP98-NUP96 precursor (77). The alternative transcript generates a precursor NUP186 protein, which is then proteolytically cleaved into NUP98 and NUP96 (77). NUP98 is encoded by the first 18 exons of the NUP98 gene and is composed of 860 amino acids, and the other exons are involved in coding the NUP96 protein (28). It is a nuclear pore protein with a molecular weight of 98 kDa, forming an important part of the nuclear pore complex (NPC) (28). Several scholars have found a dimeric or oligomeric domain in NUP98, including a coiled-coil structure (27). NUP98 plays a huge role in nucleocytoplasmic transport, allowing nucleolar proteins and RNA transporters to shuttle protein and RNA between the nucleus and cytoplasm (28).

The NUP98-FGFR1 fusion is formed from a balanced translocation, the fusion site and breakpoint of the fusion are not clear. Based on the other fusion genes mentioned above, we infer that the NUP98-FGFR1 fusion should contain a dimeric or oligomeric coiled-coil domain of NUP98. The fusion of NUP98 and other partner genes often contains the N-terminal GLFG domain of NUP98 (77), and we hypothesize that the GLFG domain contains a coiled-coil. In addition to being fused to FGFR1 on EMS, NUP98 also forms a fusion with the NSD gene on 8p11-12 due to translocation (78). Fusion transcripts of NUP98 usually have different characteristics, such as FG (phenylalanine motif), which provides a binding site for the homologous domain of karyopherins and chromatin interactions with its partner genes (44). One case presented with abdominal pain and fever, which was diagnosed as breast cancer with metastasis 11 years ago and the patient received radiotherapy and chemotherapy (79). The peripheral hemogram is mainly composed of blast cells, while the bone marrow is mostly composed of granulocyte hyperplasia with mononucleosis and a therapeutic AMML (acute monomyelocytic leukemia) phenotype (79).

### 2.6 FGFR1OP2-FGFR1/ins (8, 12) (p11.2; p11p22)

The full name of the FGFR1OP2 gene is FGFR1 oncogene partner 2, or FOP2. FOP2 is in a 12p11-12 locus with 7 exons, it encodes a protein containing 253 amino acids, approximately 29 kDa (30). As a result of a chromosome insertion, the chimeric cDNA shows an in-frame fusion of exon 4 of FOP2 to exon 9 of FGFR1, and its breakpoint is located in intron 4 of FOP2 and intron 8 of FGFR1 (30). The structure of the FOP2 protein contains four coiled-coil domains, and the first two exist in the fusion, suggesting that the fusion protein consists of 526 amino acids and is approximately 60 kDa (30). In a mouse model of the FOP2-FGFR1 fusion, the fusion protein combined with Notch1 promoted stem cells to differentiate into T cells and trigger lymphoma (80). Due to the constitutive activation of deletion mutations, the abnormal increase in Notch1 transcription in fusion T-lymphoma mice may be due to the use of an alternative Notch1 promoter (80). Notably, Hsp90 and Hsp90-CDC37 formed with the partner CDC37 could maintain the stability and activity of the FOP2-FGFR1 fusion, and Hsp90-CDC37 forms a permanent complex with FOP2-FGFR1 to protect it against hydrolysis (81). In two patients with FOP2-FGFR1, lymphadenopathy was their common clinical manifestation, their laboratory tests showed eosinophilia, and the lymph node biopsy indicated T-LBL (29, 30). It is unclear whether there is a mutation of Notch1 in patients with the T-LBL phenotype of the FOP2-FGFR1 fusion, but a mutation of Notch1 could be useful for the diagnosis and prognostication of patients in the future.

### 2.7 TIF1/TRIM24-FGFR1/t (7, 8) (q34; p11.2)

TIF1 is in 7q34 and is responsible for encoding TIF1α (transcription factor 1α). The N-terminal of TIF1α displays an RBBC motif composed of a RING finger, B-BOX, and a coiled-coil domain, also known as the tripartite motif (TRIM), and the C-terminal contains a PHD and a bromo domain (31). TIF-FGFR1 and reciprocal TIF1-FGFR1 were found in one patient. The breakpoint of TIF1-FGFR1 was in intron 9 of FGFR1 and intron 11 of TIF1 (31). The TRIM domain and tyrosine kinase domain were retained in the TIF1-FGFR1 protein, but the FGFR1-TIF1 protein was oriented to the plasma membrane by the extracellular domain and transmembrane domain of FGFR1, and the PHD and bromo domains of TIF1 and the pathogenicity of TIF1-FGFR1 were stronger than the latter (31). Nuclear receptors are ligand-induced transcription factors; currently, TIF1 is widely considered to be a protein that specifically interacts with the ligand-binding domains of several nuclear receptors (32). There are 5 known cases, three of which were found in Korea. One of the patients was not accompanied by eosinophilia at the time of onset and had increased but it subsided spontaneously (82). After nearly 5 years, eosinophilia appeared in the patient’s peripheral blood and bone marrow, and the clinical diagnosis was chronic eosinophilic leukemia. Another case had AML-M4 with eosinophilia, and one had B-lymphocytic leukemia (31, 83).

### 2.8 MY018A-FGFR1/t (8, 17) (p11.2; q25)

Myosin XVIIIA, or MYO18A, is in 17q25 and it encodes an unconventional myosin. Its N-terminal contains a PDZ domain, followed by a conserved myosin head motor domain, next to several binding sites consistent with calcitonin and calcitonin-related light chains, alias IQ motifs, and there is a coiled-coil domain included in the C-terminal (34). The PDZ domain is mainly involved in protein-protein interactions and often binds to proteins with C-terminal PDZ motifs (84). The conserved myosin motor domain plays a principal role in the interaction with ATP (34). Owing to the opposite directions of MYO18A and FGFR1 at the centromere, the MYO18A-FGFR1 fusion is not only formed by a simple chromosome balanced translocation but also involves an inversion. It is a complex FGFR1 rearrangement in which t (8, 50) is derived from a three-way translocation and accompanied by a breakpoint of 8p11 (33). The 32nd exon of MYO18A is fused to the 9th exon of FGFR1; however, it is strange that although MYO18A is in 17q11, the breakpoint of the fusion is in q23 (33). There is a reason why the distal regions of 17q23 and 8p11 are translocated to reciprocal derivative chromosomes, and then the 17q chromosome region between 17q11 and 17q23 is reversed, followed by a combination with FGFR1 on 8p11 (33).

The MYO18A-FGFR1 fusion presumably encodes a protein containing 2085 amino acids (33). The activation of the oncogenicity of the FGFR1 fusion may be closely related to the cellular localization of its partner protein. It has been reported that CSF-1(colony stimulating factor-1) could phosphorylate MYO18A, and this may change its cellular localization or affect its binding to the target protein (85). Both known cases were female patients with the CML phenotype. In one case, the peripheral blood and bone marrow showed CML-like characteristics (33). Another case developed from severe urticaria to systemic malignant mast cell disease (MCD) and was diagnosed as CML (27).

### 2.9 CPSF6-FGFR1/t (8, 12) (p11.2; q15)

Cleavage and polyadenylation specificity factor 6, also called CPSF6, is a member of the CFIm (Cleavage Factor Im complex), which plays a key role in the 3’cleavage and polyadenylation of pre-mRNA (36, 37). It is also involved in the selection of poly-A sites for multiple genes and in the regulation of the 3’UTR (36, 37). The CPSF6-FGFR1 fusion consists of exon 8 of CPSF6 fused to exon 9 of FGFR1 in-frame, and its breakpoint is in intron 8 of CPSF6 (35). The CPSF6-FGFR1 fusion mRNA is presumed to encode a protein with 895 amino acids, approximately 97 kDa, which retains the N-terminal domain of CPSF6 and contains an RRM (RNA recognition motif) (35). Nevertheless, CPSF6 has not been confirmed to have a dimerization domain and, only the RRM domain of CPSF6 is retained in the fusion, which may mediate homodimerization (35). Currently, only one case has been reported. The first episode of this patient involved lymphadenopathy and splenomegaly, and neutrophils in the peripheral blood were increased without eosinophilia, but eosinophilia was present in the bone marrow (35). Monocyte infiltration was found on lymph node biopsy.

### 2.10 LRRFIP1-FGFR1/t (2, 8) (q37; p11.2)

LRRFIP1 is a leucine-rich repeat flightless-interacting protein 1, and the LRRFIP1 protein is widely expressed in the nucleus and cytoplasm, mainly in the cytoplasm (86). In reality, LRRFIP and its function have not yet been clarified, but it participates in the regulation of the immune response. For instance, the long chain of noncoding RNA upstream of TNF binds to the inhibitor LRRFIP1, which negatively regulates the expression of TNF by forming an inhibition complex (87). LRRFIP1 and ETs-1 (ETs protein-1) interact with the TNF-α-308 site. LRRFIP1 is a TNF-α repressor that does not produce TNF-α in cells and occupies 308 sites, thus reducing TNF-α, yet the combination of ETs and the 308 site produces the opposite effect (88). Exon 9 of the two genes are fused in-frame, and the transcripts of the LRRFIP1 fusion tend to encode highly differentiated proteins that contain 668 amino acids (38). Similarly, the fusion contains the N-terminal coiled-coil domain of LRRFIP1 (38). At present, there is only one known patient, aged 82, who presented with pancytopenia (38). Five years prior, BM presented with MDS (myelodysplastic syndrome) and PB had obvious eosinophilia, and the disease turned into AML after 5 years (38).

### 2.11 CUX1-FGFR1/t (7, 8) (q22; p11.2)

CUX1 (Cut-like homeobox 1) is in 7q22, which encodes a protein that binds to DNA, and it is one of the members of the homologous domain family (homeobox transcription factors) (39). The homeobox domain and three repeated CUT domains of DNA binding form the homeobox transcription factor and the N-terminal of the protein involves a coiled-coil domain (39). The balanced translocation of chromosomes causes an in-frame fusion between exon 11 of CUX1 and exon 10 of FGFR1 (39). CUX1 is a tumor suppressor that stabilizes the PI3K signaling pathway and decreases the number of normal cells transforming into tumor cells. Previous studies using a Drosophila cancer model and a CUX1 insertion mutation mediated by a mouse transposon found that when CUX1 is deleted, it will abnormally activate the PI3K signaling pathway, thus promoting tumor growth and sensitivity to PI3K/AKT inhibitors (40). CUX1 is frequently inactivated in myeloid tumors. Knockout of the CUX1 gene promotes PI3K signal transduction, which activates quiescent hematopoietic stem cells and causes them to proliferate, leading to hematopoietic stem cell failure, causing MDS in mice (89). Otherwise, CUX1 participates in DNA repair, and CUX1 deletion leads to abnormal DNA repair, which also seems to be one of the pathogenic mechanisms of myeloid tumors. The PB of patients with CUX1-FGFR1 shows an increase in neutrophils and lymphocytes and a mild increase in monocytes without eosinophils (39). The blast cells in the peripheral blood are mainly pre-T-LBL, and the known patients died after one round of chemotherapy (39).

### 2.12 TPR-FGFR1/t (1, 8) (q25; p11,2)

**Figure 2:**
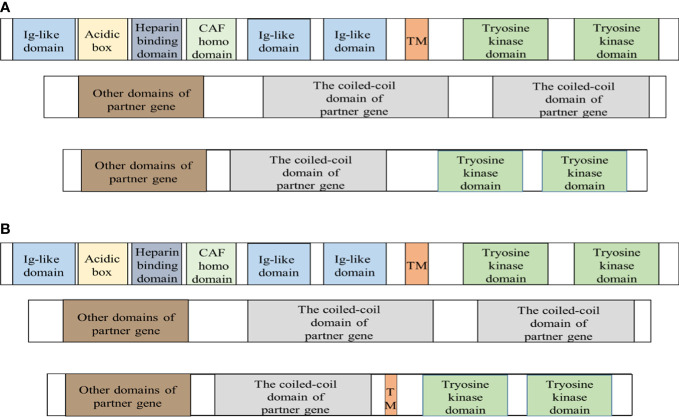
Fusion proteins are produced in two different ways. FGFR1 is a transmembrane protein and the extracellular domain of FGFR1 is a signal peptide composed of I immunoglobulin-like domain, acidic box, heparin-binding domain, and cell adhesion factor homologous domain, II immunoglobulin-like domain, and III immunoglobulin-like domain. The intracellular region consists of the tyrosine kinase domain. The proteins transcribed by the fusion gene mainly have two different protein structures. The breakpoint is indicated by the red dashed line. CAF = Cell Adhesion Factor; TM = transmembrane domain. **(A)** The fusion protein only retains the tyrosine kinase domain of FGFR1 and the coiled-coil domain of partner protein. Except for the fusion protein with unclear structure and TPR-FGFR1, others are basically of it. **(B)** TPR-FGFR1 not only contains the tyrosine kinase domain of FGFR1 and a variety of the coiled-coil domain of TPR but also retains the partial transmembrane domain of FGFR1.

TPR is also known as the translocated promoter region, it is in 1q25 and consists of 51 exons. The mammalian TPR encodes a nuclear pore protein including 2349 amino acids of approximately 267 kDa (43). The N-terminal residues include the TprMet domain, NPC relevant domain, and multiple coiled-coil domains, followed by several leucine zipper domains and phosphorylated sequences of various kinases. The C-terminal is composed of a highly acidic spherical domain (90, 91). TPR is located in the nucleoplasmic fibrils of NPCs, which are located on the cytoplasmic surface of the nuclear membrane (91). Comparison between the cDNA sequence and the genomic DNA sequence revealed that the chimeric cDNA of TPR–FGFR1 is the result of an in-frame fusion of exon 22/23 of the TPR gene to exon 13 of FGFR1, and the breakpoint is in intron 22 of TPR and intron 12 of FGFR1 (41, 92). The chimeric protein includes 1426 amino acids of approximately 154 kDa, and it exists in the cytoplasm and contains the TprMet domain, NPC relevant domain, multiple coiled-coil domains, tyrosine kinase domain, and partial transmembrane domain of FGFR1. There are two different fusion ways for other fusion proteins and TPR-FGFR1 fusion protein (**Figure 2**).The TPR part has one or more dimerization domains (41, 42). In addition, dimerization may be provided by the TPR to guide the fusion to the NPC, and there is a dramatic influence on the regulation of nucleocytoplasmic transport and the molecules entering or leaving the nucleus due to the localization of the fusion protein kinase (90). There are four known cases with TPR-FGFR1. Lymphadenopathy was the common manifestation at onset, and lymphoid biopsy found was T-cell lymphoma. PB and BM had increasing monocytes, with or without eosinophilia, and the bone marrow was MPN(myeloproliferative neoplasms)-like (92). Finally, the clinical phenotype was diagnosed as AMML and AML-M5 (41).

### 2.13 NUP358/RANBP2-FGFR1/t (2, 8) (q12; p11.2)

NUP358 is at 2q12, and it encodes a polypeptide chain by containing 3224 amino acids of 358 kDa (45), also called RANBP2. NUP358 could approximately be divided into several distinct regions: The N-terminal of NUP358 contains an α-helical region (which has three nonstandard tetratricopeptide repeats (TPRs) with the property of right-handed torsion), followed by four RanGTP binding domains, eight consecutive zinc finger motifs, an E3 ligase domain, and a C-terminal cyclophilin A homology domain (they are presumed to be connected to an unstructured region containing phenylalanine glycine (FG) repeats, forming the docking site of the mobile transport receptor) (45). NUP358, like NUP98, is an important component of the NPC and it plays a major role in transport between the nucleus and cytoplasm. Located on the cytoplasmic surface of NPC, NUP358 has a high-intensity positive charge and it binds to single-stranded RNA and exports RNA as a major part of the nuclear-cytoplasmic transport process (45). With a balanced translocation, exon 20 of NUP358 fused to exon 9 of FGFR1, and the fusion is predicted to encode a chimeric protein containing RANBP2 as the N-terminal, as well as a leucine zipper part that mediates protein-protein interactions (44). The only known patient was a 63-year-old woman with splenomegaly and a little eosinophilia (44). Granulocytes were the main features of peripheral blood and bone marrow biopsy, mainly manifesting as MDS (myelodysplastic syndrome) (44).

### 2.14 HERVK-FGFR1/t (8, 19) (p11.2; q13)

Human endogenous retrovirus-K (HERVK) is a residue from being infected by a retrovirus and integrating into the human genome, and it can be divided into three families: class I, class II and class III. Among them, HERVK belongs to class IIβ retroviral-like elements and is also called the HERVK superfamily (93). The expression of HERVK family proteins can trigger the proliferation and transformation of normal cells, especially into leukemia cells. Unfortunately, due to the lack of research on its rearrangement with FGFR1, more molecular information cannot be provided. An EMS patient with HERVK-FGFR1 fusion had systemic mastocytosis, and KIT and D815V mutations could be detected (similar to case 8p11 complicated with mastocytosis, MYO18A-FGFR1, and ZNF198-FGFR1). There was a common characteristic that both had erythroid abnormalities: one had polycythemia and poikilocytes in the PB (94), and the other had erythroid maturation disorder (22). Furthermore, two cases also had granulocytosis and abnormal megakaryocytes; one case was diagnosed as AML, and the other was diagnosed as systemic mastocytosis with clonal hematopoietic nonmast cell disease (SM-AHNMD) (22, 94). The fusion protein of HERVK-FGFR1 may the development and maturation of erythroid and megakaryocytes, which requires further research to provide more evidence.

### 2.15 SQSTM1-FGFR1/t (5, 8) (q35; p11.2)

SQSTMI at 5q35 is composed of 8 exons and encodes a multifunctional protein with 440 amino acids and a molecular weight of 62 kDa, so it was also previously called p62. This protein binds to ubiquitin and regulates the activation of the NF-κβ signaling pathway, which is closely related to oxidative stress and autophagy (46, 47). The structural skeleton of SQSTM1 is the N-terminal of the PB1 (Phox and Bem1p) domain, ZZ type zinc finger domain, LIR (LC3 interaction domain) motif, and the C-terminal of UBA (ubiquitin associated) domain, which mediates the interaction with single or multiple ubiquitins (95, 96). Mutation of the UBA domain is associated with Page’s disease of the bone (PDB) (95, 96). The PB1 domain of SQSTM1 mediates the homodimerization of SQSTM1 through the electrostatic force interaction between alkaline and acidic charge clusters at appropriate positions, in which the alkaline charge cluster plays a key role (96). Sequencing of the PCR product revealed that FGFR1 was fused to exon 9 of SQSTM1 at chromosome 5q35, showing that SQSTM1 was juxtaposed with FGFR1 as a result of chromosomal translocation and that the breakpoint was intron 8 of SQSTM1 and intron 8 of FGFR1 (46). The transcript of the fusion presumably encodes a protein containing 718 amino acids, and the N-terminal retains the PB1 domain of SQSTM1, thus enabling cell transformation (46). The activation of the constitutive tyrosine kinase SQSTM1-FGFR1 may be mainly due to its homodimerization mediated by acid and alkaline charge cluster interactions. In this case, neutrophils and monocytes were increased in the peripheral blood, but the bone marrow was dominated by monocytes and megakaryocytes. There was only one case of SQSTM1-FGFR1 without eosinophilia that was diagnosed as AMML (46).

### 2.16 TFG-FGFR1/t (3, 8) (q12; p11.2)

TFG is at 3q12, also known as the tropomyosin-receptor kinase fused gene or TRK fusion gene, and it was first discovered as a fusion partner of NTRK1 in human papillary thyroid carcinoma (97). The coiled-coil domain of TFG is made up of four leucine motifs with a heptapeptide repeat region, which might be the reason why it is shorter than the typical leucine zipper (98). The human TFG protein sequence is highly homologous to that in pigs and mice, and the structure of human TFG contains a coiled-coil domain of the N-terminal trimer, glycosylation, myristylation and phosphorylation region, and the SH2- and SH3-binding motifs (97). The specificity of TFG dimerization may be due to changes in the Val and Leu residues at core position A (98). The TFG-FGFR1 fusion site is not clear, but the breakpoint is in exon 8 of TFG and exon 10 of FGFR1 (48). The fusion encodes a protein located within the cytoplasm, and the results of *in vitro* coimmunoprecipitation showed that the fusion could self-bind to form a homodimer (48). The TFG-FGFR1 fusion activates the downstream cascade signaling pathway in cells and regulates the FGFR1 downstream genes, which promotes the upregulation of BCL2, MYC, and KLF4 expression and the downregulation of SPI1 and CSFIR expression, and the fusion regulates downstream signaling pathways, mainly *via* SPI1 (48). Furthermore, the upregulation of MYC by the fusion leads to the phosphorylation of STAT3, STAT5, ERK, FLT3, and JNK, promoting the continuous proliferation of cells (48). The one known patient suffered from skin ecchymosis and splenomegaly, and eosinophilia was not found in PB and BM examination (48). The patient was diagnosed with AML with maturation; later, as with most patients with EMS of other subtypes, he died because of conventional chemotherapy (48).

### 2.17 HOOK3-FGFR1/inv (8, 8) (p11.23; p11.21)

HOOK3-FGFR1 positivity with EMS is the latest FGFR1 rearrangement discovered, more importantly, the reason for its formation is different from that of its other partner genes. It is an inversion forming a ring chromosome 8 but the existence of the ring chromosome is related to genomic instability (49). The breakpoints of the fusion are in exon 11 of HOOK3 and exon 10 of FGFR1, and it is speculated that the chimeric protein contains 768 amino acids (49). As expected, the N-terminus of the fusion contains a partial coiled-coil domain encoded by exons 31-11 of HOOK3, and the C-terminus contains a complete tyrosine kinase domain encoded by exons 10-18 of FGFR1, excluding the transmembrane domain (49). According to recent studies, the activation of the NF-κβ pathway is an important factor in the treatment of multiple myeloma and is associated with low sensitivity to bortezomib and ixazomib (99). Only one case of HOOK3-FGFR1 has been reported. The patient was admitted to the hospital with nonspecific clinical symptoms, the PB showed leukocytosis and thrombocytopenia, and BM revealed myelodysplasia and B-lymphoid and granulocytic infiltrative hyperplasia without eosinophilia (49). Ultimately, the patient was clinically diagnosed with MDS with abnormal monoclonal B-cell proliferation (49).

## 3 Targeted therapy with tyrosine kinase inhibitors

To date, the prognosis of EMS is very unfavorable. Few cases have achieved remission, and most of them were treated by allogeneic hematopoietic stem cell transplantation (HSCT). Notwithstanding that a variety of traditional chemotherapy regimens were also involved in the treatment, the outcomes were not satisfactory, and serious side effects and drug resistance resulted in undesirable events for the patients. Additionally, because of the scarcity of stem cell donors, difficulties in matching, infection, economic costs, and a long waiting time, the majority would die of disease progression before HSCT. It was exciting that in recent years, the first TKI, imatinib, was developed and applied to the treatment of CML (Philadelphia chromosome-positive), and an excellent effect was achieved. Then, a variety of TKIs was developed and tested in clinical trials, which brought hope to patients. FGFR1 rearrangement leads to the constitutive activation of a tyrosine kinase, which triggers cascade signal transduction in cells and causes abnormal proliferation, survival, differentiation, and antiapoptotic effects. Therefore, inhibitors targeting tyrosine kinase have the potential for the treatment of EMS. There are the number and the reported response for chemotherapy or TKIs (**Table 2**).

### 3.1 Mechanism

There are various types of tyrosine kinase inhibitors; in general, their mechanisms are mainly the following:

1. Small molecule TKIs target the ATP-binding cleft in growth factor receptor kinases (100);

2. Peptide inhibitors of pseudosubstrates that bind to catalytic domain peptide/protein substrate sites (101);

3. Monoclonal antibodies against receptor tyrosine kinase and ligand traps (101, 102).

Small molecule inhibitors account for the majority of TKIs, which are commonly used to treat lung cancer, breast cancer, and other tumors; in particular, the efficacy of inhibitors targeting VFGFRs has been quite apparent during treatment. Small molecule inhibitors are mostly ATP-competitive inhibitors that compete with ATP and combine with the TK receptor kinase cleft to inhibit kinase activity and its downstream intracellular signal cascade reactions, thus inhibiting the proliferation and transformation potential of oncogenes (100). All protein kinases share the same ATP binding site, and the binding of ATP to a kinase is due to the hydrogen bond between the adenine ring of ATP and the ATP binding cleft of the kinase. The inhibitors target the ATP binding site of the kinase and vicinity and the selectivity of inhibitors is controlled by simulating different parts of the ATP structure (103).

**Figure 3:**
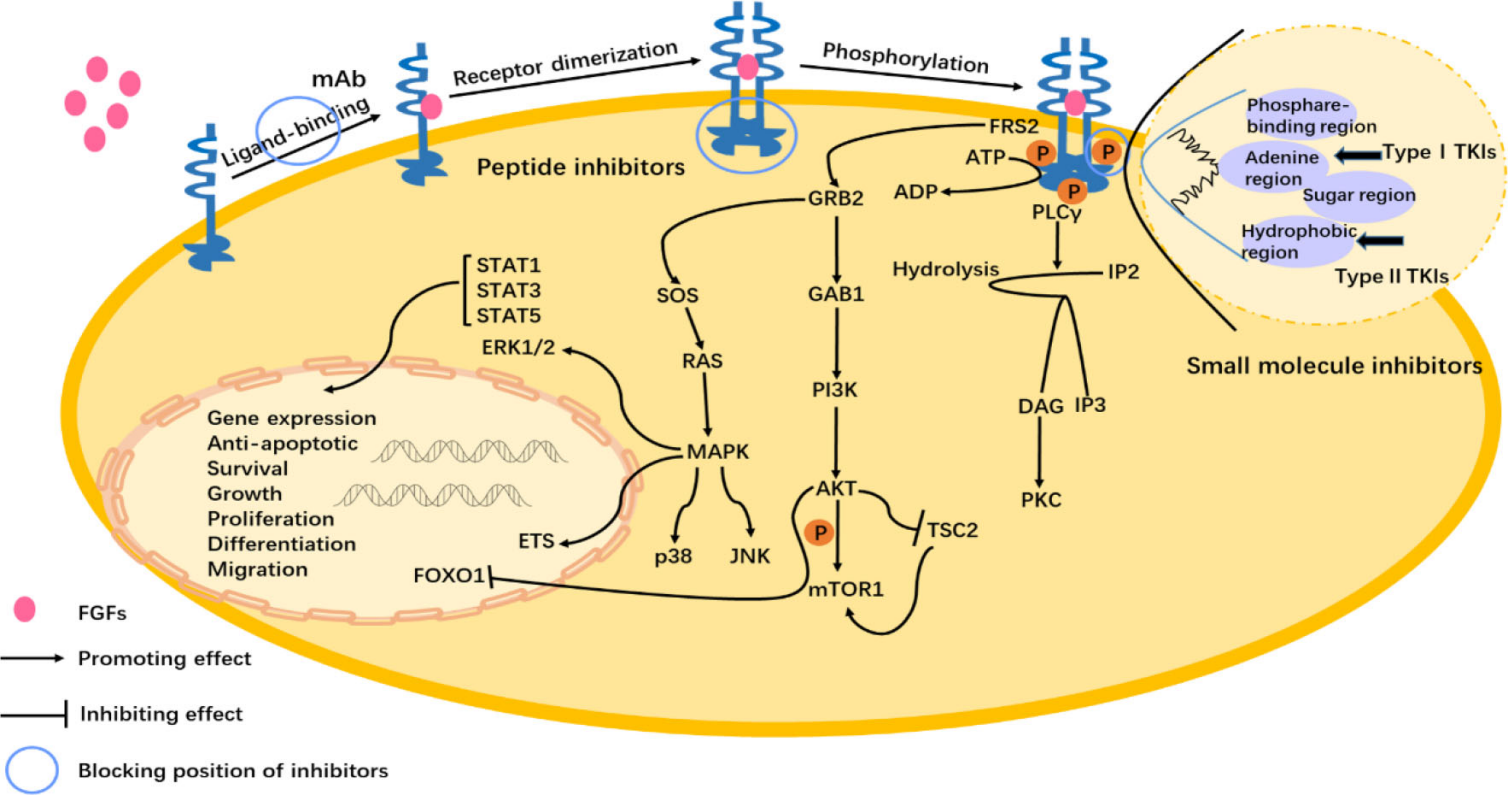
FGFs/FGFR1 signal pathway and the mechanisms of FGFR1 inhibitors. FGFs binding to the FGFR1 induces dimerization and the subsequent phosphorylation of the intracellular tyrosine kinase domain. Activation of downstream signaling occurs via FGFR substrate 2α (FRS2α), which is constitutively associated with the juxtamembrane region of FGFR. Phosphorylated FRS2 recruits the adaptor protein, growth factor receptor-bound protein 2 (GRB2), which then recruits the guanine nucleotide exchange factor SOS. The recruited SOS activates the RAS GTPase, which then activates the MAPK pathway. MAPK activates members of the ETS transcription factor family, ERK1/2, p38, and Jun N-terminal kinase pathways (JNK). The recruited GRB2 as well as recruits the adaptor protein GAB1, which then activates the PI3K, and after that phosphorylates the AKT. Next, then AKT has various activities including activation of the mTOR complex 1 by inhibition of the cytosolic tuberous sclerosis complex 2 (TSC2) and phosphorylation. And AKT pathway inhibits the activity of the forkhead box class transcription factor (FOXO1) bringing about it exiting the nucleus. Phospholipase C (PLC) binds to a phosphotyrosine and hydrolyzes phosphatidylinositol 4,5-bisphosphate (IP2) to phosphatidylinositol 3,4,5-triphosphate (IP3) and diacylglycerol (DAG), which then activates protein kinase C (PKC). The target gene expression is regulated by the activity of the signal transducer and activator of transcription STAT1 (signal transducer and activator of transcription), STAT3, and STAT5. The abnormal activation of the FGFs/FGFR pathway has an influence on physiological activity, such as anti-apoptotic, survival, and growth of cells (1–3). The mechanisms have been shown in the figure of small molecule inhibitors, peptide inhibitors, and monoclonal antibodies.

ATP small molecule inhibitors are divided into three types, I, II, and III. Type I inhibitors recognize the active conformation of kinases and are direct ATP competitive inhibitors. Inhibitors form one to three hydrogen bonds with the binding sites of kinases, simulating the formation of hydrogen bonds between normal ATP and binding sites, competing with ATP for ATP binding sites (104). Conversely, the type II inhibitor work by binding to the inactive conformation of the kinase, and it is an ATP indirect competition inhibitor that allosterically regulates the kinase activity and occupies the hydrophobic sac adjacent to the ATP binding site, and then indirectly competes with ATP (105). Different from the first two, type III inhibitors are covalent inhibitors that covalently bind to a cysteine on particular parts of the kinase. The sulfur atom in the cysteine residue is rich in electrons and reacts with the electrophilic group of the inhibitor, sharing electrons and irreversibly binding, blocking the kinase from binding to ATP (106). Covalent inhibitor binding can occur at any variable position of cysteine residues in the kinase domain, so that position is not fixed (103). FGFs/FGFR1 signal pathway and the mechanisms of FGFR1 inhibitors (**Figure 3**).

It has been pointed out in the available literature that TKIs used in EMS treatment are small molecule inhibitors of ATP-binding clefts, but they are classified as nonselective or selective depending on their targeting selectivity to FGFR. The former mentions targeting a variety of growth factor receptors, including FGFRs, which are multitargeted; the latter refers to targeting only FGFRs or targeting a variety of growth factors with the highest affinity for FGFRs.

### 3.2 Nonselective tyrosine kinase inhibitors

PTKs (protein tyrosine kinases) play an important role in cell regulation, such as mitosis, development and differentiation, tumorigenesis, angiogenesis, cell survival and apoptosis, and cell cycle control. Therefore, abnormal PTKs interfere with normal physiological functions, thus promoting the occurrence and development of diseases and even tumors (100, 107). FGFRs, VFGFRs, and PDGFRs are members of the PTK superfamily, and their protein structures are similar to those containing the extracellular ligand-binding domain, the transmembrane domain, and the intracellular tyrosine kinase domain. Their tyrosine kinase domains are highly similar, especially their ATP binding sites (103). Therefore, small molecule ATP inhibitors target a variety of growth factors with similar kinase domains and are also known as multitarget TK inhibitors, nonselective.

#### 3.2.1 Imatinib

Imatinib was approved by the FDA to treat Philadelphia chromosome-positive CML in 2001, and encouraging data were obtained in subsequent clinical trials (108). It is a competitive inhibitor of ATP type II and is also approved for treating some solid tumors (109, 110). Although the application of imatinib can reduce the blood cell count, no reliable data have been obtained *in vitro*, and its actual therapeutic effect on EMS is unclear. One case presented with 8p11 and ZMYM2-FGFR1 fusion positivity, and the patient then began taking imatinib on the 15th day after diagnosis (111). Although the leucocyte count decreased briefly and returned to normal, the patient unfortunately died due to disease progression (111). Disappointingly, the results obtained with imatinib *in vitro* were unsatisfactory. Imatinib could not inhibit FGFR1 rearrangement well in cell lines compared to other small molecule inhibitors (112, 113). Imatinib might have a therapeutic effect on the chronic phase of the CML phenotype in EMS, for which the main treatment is to reduce the leucocyte count in the patients’ PB, but it may have little benefit for the treatment of EMS in the acute phase.

#### 3.2.2 Ponatinib

Ponatinib (AP24534) is an oral multitarget inhibitor that mainly targets ABL, FGFR1, FLT3, KIT, and PDGFRA (114). The application of ponatinib in EMS has also been supported by many researchers and clinicians. Ponatinib showed agreeable direction on a variety of EMS cell lines *in vitro*, mainly by inhibiting the downstream signaling pathways of ERK and STAT5 and inducing cell apoptosis (115). A 47-year-old male patient with BCR-FGFR1 positivity was planned to receive single-agent ponatinib due to the ineffectiveness of MEC chemotherapy at the beginning (57). The patient had good tolerance to the treatment and achieved satisfactory results, with the resolution of his swollen lymph nodes and neck pain, and the morphology of his BM was completely restored, providing an opportunity to implement hematopoietic stem cell transplantation (57). Attention to the NF-κβ pathway in FGFR1 rearrangement and further enrichment may be one of the reasons for the poor response to ponatinib (49). There is a presumption that ponatinib treatment can achieve more survival opportunities for patients who are resistant to EMS traditional chemotherapy and imatinib with lymphadenopathy, establish clinical remission, and improve the quality of life of the patients.

#### 3.2.3 Midostaurin

Midostaurin is a small molecule tyrosine kinase inhibitor that is mostly involved in the treatment of AML and advanced systemic mastocytosis. Midostaurin demonstrated favorable results in ZNF198-FGFR1 mouse models and cells. The Midostaurin-treated group survived significantly longer and the spleen weight, and white blood cell count was significantly lower than the placebo-treated group (116). Based on the above data, a patient with ZNF198-FGFR1 enrolled in a randomized phase II clinical trial and obtained a nice treatment response. His lymphadenopathy and splenomegaly significantly subsided, and the patient achieved clinical stability for 6 months (116). This evidence suggests that Midostaurin can be effective for patients with a progressive myeloproliferative disorder with organ enlargement.

#### 3.2.4 Dovitinib

Dovitinib (TKI258) is a multitarget receptor tyrosine kinase inhibitor targeting FGFRs, VEGFR, PDGFR, FLT3, and KIT. TKI258 inhibited the phosphorylation levels of ERK and STAT5 in cells transformed by the fusion in a dose-dependent manner (117). There was a significant difference in the efficacy of dovitinib between the CFU-GM (CFU-granulocyte-macrophage) and BFU-Es (erythrocyte burst-forming unit). CFU-GM was not inhibited by dovitinib, but BFU-Es were strongly inhibited at all below 100nM (117). In addition, the *in vitro* data of dovitinib compared with other TKIs are not ideal, and larger doses are required to achieve the same degree of inhibitory effect as other TKIs (118). The difference in efficacy between dovitinib and other TKIs may be related to the non-selective expression level of this kinase and the inhibition of the number of other potential kinases and downstream biological efficacy. This suggests that colony FISH analysis is very important before the treatment of actual EMS patients, which could determine whether dovitinib could reduce the number of FGFR1 rearrangement subtype cells.

#### 3.2.5 Dasatinib

Dasatinib is an oral small molecule inhibitor that inhibits nonmutated BCR-ABL and most known BCR-ABL mutants; its targeting includes PDGFR, cKIT, SFK (SRC family kinase), FGFR1, and EGFR (114). Several clinical examples suggest that dasatinib is more suitable for EMS patients with cardiovascular disease than ponatinib, and the patients benefited for more than 9 months and significantly improved the patient’s quality of life (118, 119). Perhaps dasatinib might be more suitable for the treatment of elderly and frail EMS patients with cardiovascular disease, and it improves the PB, prolongs the survival time of patients, and provides another treatment option in addition to hematopoietic stem cell transplantation.

### 3.3 Selective tyrosine kinase inhibitors

Even though nonselective TKIs have shown their possible efficacy in EMS, their side effects should not be ignored due to their multiple targets. To enhance the efficacy and reduce the side effects, it is necessary to selectively block the FGFR tyrosine kinase. Recently, with the development of selective FGFR inhibitors such as pemigatinib and infigratinib, phase I/II clinical trials have been carried out for treating advanced cholangiocarcinoma, and they may also have potential therapeutic effects for EMS.

#### 3.3.1 Pemigatinib

Pemigatinib (INCB054828) is a reversible ATP competitive FGFR inhibitor, and the US FDA has accelerated the approval of pemigatinib for the treatment of previously treated and unresectable locally advanced or metastatic cholangiocarcinoma with FGFR2 fusion or other rearrangements (120). In August 2019, pemigatinib was recognized as an orphan drug in the US to treat myeloid/lymphoid tumors with eosinophilia, PDGFRA, PDGFRB, FGFR1 rearrangement, or PCM1-JAK2. In enzymatic assays with recombinant human FGFR kinases, the IC_50 values_ of INCB054828 against FGFR1, 2, and 3 were 0.4, 0.5, and 1.0 nM, respectively, but the inhibitory effect on FGFR4 was weak (121). It is speculated that its high affinity and selectivity for FGFR1, FGFR2, and FGFR3 are due to its filling with complementary hydrophobic vesicles near the FGFR gatekeeper region (120).

Pemigatinib has been studied in several EMS registered clinical trials and *in vitro* trials, with good tolerance and excellent efficacy. *In vitro*, pemigatinib could effectively inhibit FGFR phosphorylation, pERK, and pSTAT5 levels, and restore FGFR phosphorylation to basal levels (121, 122). In addition, humanized mice subcutaneously transplanted with KG1 could significantly inhibit the growth of tumors by oral administration of pemigatinib, and phase II clinical trials have been carried out in FGFR1 rearranged myeloid/lymphoid tumors (FIGHT, NCT03011372) (121). Thirty-four MLN patients were included in the trial, and the results showed that among 31 patients with BM and/or involvement of extramedullary disease (EMD), the CR (complete response) rate and CRC (clinical research coordinator) assessment were 64.5%, and 77.4%, respectively (123). Among the 33 patients evaluable for CyR (cytogenetic responses), the CCyR (complete cytogenetic response) rates were 72.7% and 75.8, respectively, but the median CR duration had not been reached (123). Recently, these results suggest that pemigatinib may offer a long-term treatment option for EMS ineligible for HSCT or may facilitate bridging to HSCT in eligible patients.

#### 3.3.2 Infigratinib

Infigratinib (NVP-BGJ398) is a selective and oral small molecule FGFR inhibitor. Different from non-selective TKIs, BGJ398 could significantly inhibit the expression of the FOP2-FGFR1 fusion protein, apoptosis-related protein BCL-2, and phosphorylation levels of AKT and S6K1, and upregulate activated caspase-3 (117). Apoptosis is a complex process regulated by many genes, and the regulation of apoptosis-related proteins by BGJ398 may be one of the mechanisms by which it exerts its pharmacological actions (117). What’s more, Other studies have confirmed that both ponatinib and infigratinib can inhibit the proliferation of TPR-FGFR1 fusion protein, but the effect of infigratinib is stronger than ponatinib, and infigratinib induces the death of transformed cells at a very low concentration (42).

#### 3.3.3 Other potential inhibitors

In addition to pemigatinib and infigratinib, the following FGFR inhibitors also have the potential to treat EMS. Futibatinib is an oral, potent, selective, covalently irreversible small molecule inhibitor that targets the P-loop ATP binding pocket of the tyrosine kinase domain in FGFR1-4 *in vivo* (124–126). Futibatinib effectively inhibits FGFR1-4 with an IC_50_ of a single digit of nanomolesper liter (124–126). Erdafitinib is a new pan-FGFR small molecule inhibitor. It has recently been approved for patients with advanced urothelial cancer with specific FGFR gene changes (127). At present, there is no corresponding clinical trial or *in vitro* experiment to confirm its efficacy in rare diseases such as EMS. It is hoped that additional trials will be conducted in the future to enrich the treatment options, promote the development of FGFR small molecule inhibitors for EMS treatment and prolong the overall survival rate of these patients without requiring hematopoietic stem cell transplantation.

## 4 Conclusion

From 1998 to 2022, 17 genotypes of EMS phenotypes with FGFR1 rearrangement were reported. In recent years, because of the progress of molecular detection technology and the expansion of the detection range, the rearrangement of FGFR1 has received attention, and new mutations have been detected. Moreover, some of these fusions are not common and are only reported in a few cases, which limits the possibility of making conclusions about these new fusions. Although FGFR1 has different rearrangements; and its fusion proteins have different structures, the main functional abnormality is always caused by the FGFR1 tyrosine kinase domain in the fusion. In the future, it could be possible to conduct in-depth research on these rare fusions, including their pathological and cellular biochemical characteristics, to provide a more extensive research base to support better treatment, such as a combination of traditional chemotherapy and targeted drugs, a combination of targeted drugs and hematopoietic stem cell transplantation, or the application of targeted drugs alone. In addition, many researchers believe that 8p11 is a leukemia stem cell cancer with the potential to differentiate into various lineages, which provides strong evidence for which hematopoietic stem cells from healthy humans are the source of leukemia and the clinical relevance of the identification of pre-leukemia HSCs (128). Because of the late diagnosis and rapid deterioration of the condition, most patients with EMS have a poor prognosis and even face recurrence after transplantation, and the treatment of these patients is challenging. More effective experiments *in vitro* and clinical trials registered in centers or multiple institutions are required in the future, but they might have to be limited to rare cases.

In summary, EMS is very rare, and its clinical features are not yet very clear. Such diseases are often resistant to traditional chemotherapy, and the application of TKIs is promising for EMS treatment. In particular, attention should be given to clinical trials of FGFR inhibitors in the future.

## Author contributions

QL: Conceptualization, supervision. TL: Data curation, writing- original draft preparation. GZ: Data curation, visualization. HL: Data curation, investigation. XZ: Investigation, methodology. QL: Writing- review and editing. All authors have read and agreed to the published version of the manuscript. All authors contributed to the article and approved the submitted version.

## Funding

The work is supported by research funding from the National Natural Science Foundation of China (81302860 to QL), Science and Technology Department (20180101132JC to QL), and Finance Department of Jilin Province, China (JLSWSRCZX2020-055 to QL).

## Conflict of interest

The authors declare that the research was conducted in the absence of any commercial or financial relationships that could be construed as a potential conflict of interest.

## Publisher’s note

All claims expressed in this article are solely those of the authors and do not necessarily represent those of their affiliated organizations, or those of the publisher, the editors and the reviewers. Any product that may be evaluated in this article, or claim that may be made by its manufacturer, is not guaranteed or endorsed by the publisher.
